# TRAJECTORIES OF FORMAL SOCIAL PARTICIPATION, GENDER, AND END-OF-LIFE CARE QUALITY AMONG OLDER AMERICANS

**DOI:** 10.1093/geroni/igad104.0189

**Published:** 2023-12-21

**Authors:** Kafayat Mahmoud, Jarron Saint Onge, David Ekerdt

**Affiliations:** Boston University, Boston, Massachusetts, United States; University of Kansas, Lawrence, Kansas, United States; University of Kansas, Lawrence, Kansas, United States

## Abstract

Limited research has examined the association between formal social participation trajectories and end-of-life care quality. The end of life could be characterized by experiences of heightened feelings of physical and psychological distress, breathlessness, constant hospitalization, and intrusive interventions. Formal social participation may improve the end-of-life care of older adults because they serve as sources of useful information, receipt of emotional support, and improve self-efficacy. This research examines the associations between formal social participation trajectories and proxy ratings of overall end-of-life care quality, and the moderating role of gender. Growth-based trajectory models were used to identify distinct developmental trajectories of formal social participation among older adults in the United States. Findings revealed four social participation trajectory classes among older adults towards the end of life, all with a general tendency to decline across time. Multinomial logistic regression analyses showed that although older adults with higher levels of formal social participation have more positive overall end-of-life care ratings, there are gender differences in these care ratings. Women are less likely than men to chart, by proxy report, positive care ratings at the end of life even though they have higher levels of formal social participation, and these gender differences in end-of-life care rating are explained more by healthcare factors than formal social participation trajectories. These results suggest that both formal social participation and positive interactions with health care at the end of life are more beneficial for older men than women.

